# Analytical Solutions of Fractional-Order Diffusion Equations by Natural Transform Decomposition Method

**DOI:** 10.3390/e21060557

**Published:** 2019-06-03

**Authors:** Rasool Shah, Hassan Khan, Saima Mustafa, Poom Kumam, Muhammad Arif

**Affiliations:** 1 Department of Mathematics, Abdul Wali khan University, Mardan 23200, Pakistan; 2 Department of Mathematics, Pir Mehr Ali Shah Arid Agriculture University, Rawalpindi 46000, Pakistan; 3 Center of Excellence in Theoretical and Computational Science (TaCS-CoE) & Department of Mathematics, Faculty of Science, King Mongkuts University of Technology Thonburi (KMUTT), 126 Pracha Uthit Rd., Bang Mod, Thung Khru, Bangkok 10140, Thailand; 4 Department of Medical Research, China Medical University Hospital, China Medical University, Taichung 40402, Taiwan

**Keywords:** Natural transform decomposition method, fractional-order of diffusion equations, Mittag–Leffler function

## Abstract

In the present article, fractional-order diffusion equations are solved using the Natural transform decomposition method. The series form solutions are obtained for fractional-order diffusion equations using the proposed method. Some numerical examples are presented to understand the procedure of the Natural transform decomposition method. The Natural transform decomposition method has shown the least volume of calculations and a high rate of convergence compared to other analytical techniques, the proposed method can also be easily applied to other non-linear problems. Therefore, the Natural transform decomposition method is considered to be one of the best analytical technique, to solve fractional-order linear and non-linear partial deferential equations, particularly fractional-order diffusion equation.

## 1. Introduction

The idea of fractional calculus and entropy are attractive and more prevalent for investigating the dynamics of complex systems. In modern years, fractional calculus (FC) has been progressively applied in various fields of science. Natural development identified with viscoelasticity, models of porous electrodes, thermal stresses, electromagnetism, propagation of energy in dissipative systems, relaxation vibrations, and thermoelasticity are effectively portrayed by fractional differential equations (FDE’s) [1]. The knowledge of entropy was presented in the field of thermodynamics by Clausius (1862) and Boltzmann (1896) and was further applied by Shannon (1948) and Jaynes (1957) in information theory. Newly, more universal entropy measures have being suggested for applications in numerous varieties of complex systems, outstanding to the relaxation of the additive axiom [2]. The idea of entropy for calculating the dynamics of multi-particle systems with integer- and fractional-order behavior was suggested in [3]. The entropy production rate for the fractional diffusion procedure was considered in [4]. In [5], it has been shown that the total spectral entropy can be used as a measure of the data, comfortable in a fractional-order model of anomalous diffusion. Entropies based on fractional calculus [6]. Feng’s first integral method was applied successfully to obtain nonlinear space-time fractional modified Korteweg–de Vries equations [7], nonlinear partial differential equations, third-order dispersion [8,9] in entropy and convexity, fractional derivative advection-diffusion in two-dimensional semi-conductor systems, and the dynamics of a national soccer league [10]. The exact solution to differential equations (DEs) of fractional-order with mixed partial derivatives [11] and space-fractional diffusion equation and Tsallis relative entropy [12]. Diffusion forms contrast from regular diffusion in that the scattering of particles continues quicker (super diffusion) or slower (sub diffusion) than for the ordinary case.

Adolf Fick described Fick’s laws of diffusion in 1885. After that, Fick’s second law became known as the diffusion equation. Diffusion is the mesh movement of atoms or molecules from an area of higher concentration or great chemical potential to an area of inferior concentration or small chemical potential. In [13], the researchers generalized the classical diffusion and wave equations—different physical process such as classical diffusion, slow diffusion, the classical wave equation, and diffusion-wave hybrid. Many applications of diffusion equation, such as electrochemistry, phase transition, filtration, electromagnetism, acoustics, biochemistry, cosmology, and dynamics of biological groups [14]. Diffusion is determined by a gradient in chemical potential of the diffusing types. A gradient is the variation in the value of a number, e.g., concentration, pressure, or temperature with the variation in one or more variables being frequently distinct. A variation in temperature ended with a distance is called a temperature gradient, a variation in concentration over a distance is called a concentration gradient, and a variation in pressure ended with a distance is called a pressure gradient. Scientists have been attempting to comprehend and diminish the challenges of industrial procedures to accomplish higher effectiveness [15]. In engineering systems, there are different causes for entropy generation. In thermal systems, the primary source of entropy generation is mass transfer, heat transfer, viscous dissipation, coupling among heat, electrical conduction, and chemical reaction, as examined in a pioneering series of publications by Bejan and co-workers [16,17]. Researchers have used various techniques for the solution of diffusion equations such as the Collocation method (CM) [18], Diffusion and Tsallis entropy [19], Entropy production, Symmetric fractional diffusion [20], Finite differences method in space-fractional diffusion equations [21,22,23], Homotopy analysis method (HAM) [24], Homotopy perturbation transform method (HPTM) [25] and Modified homotopy perturbation method (MHPM) [26], Mehshless method (MM) [27], One-Dimensional alpha fractional diffusion [28], Radial basis function method (RBFM) [29], and the Variational iteration method (VIM) [30].

In the present work, we are applying the Natural transform decomposition method (NTDM) to solve the following types of diffusion equations.

(1) Two-dimensional fractional-order diffusion equation of the form:
  $$\frac{\partial^{\gamma }\upsilon }{\partial t_{1}^{\gamma }}=\frac{\partial^{2}\upsilon }{\partial x_{1}^{2}}+\frac{\partial^{2}\upsilon }{\partial y_{1}^{2}},\;0<\gamma \leq 1,\;t_{1}\geq 0,$$
  subject to the initial condition
  $$\upsilon \left(x_{1},y_{1},0\right)=g\left(x_{1},y_{1}\right).$$
(2) Three-dimensional fractional-order diffusion equation is given by
  $$\frac{\partial^{\gamma }\upsilon }{\partial t_{1}^{\gamma }}=\frac{\partial^{2}\upsilon }{\partial x_{1}^{2}}+\frac{\partial^{2}\upsilon }{\partial y_{1}^{2}}+\frac{\partial^{2}\upsilon }{\partial z_{1}^{2}},\;0<\gamma \leq 1,\;t_{1}\geq 0,$$
  subject to the initial condition
  $$\upsilon \left(x_{1},y_{1},z_{1},0\right)=g\left(x_{1},y_{1},z_{1}\right).$$

Natural transform and the Adomain decomposition method are two powerful methods that have been used to develop the Natural transform decomposition method. Many physical phenomena that are modeled by PDEs and FPDEs are solved using NTDM, such as the analytical solution of a coupled system of non-linear PDEs, are suggested in [31], the solution non-linear ODEs are successfully presented in [32], non-linear PDEs in [33], fractional unsteady flow of a polytropic gas model in [34], fractional telegraph equations in [35], and the fractional Fokker–Plank equation and Schrödinger equation in [36]. The accuracy of the proposed method is compared with the solutions obtained by HPM and MHPM. The comparisons have shown that the proposed method has a higher rate of convergence than HPM and MHPM. The rest of the article is structured as follows: In Section 2, we recall several basic properties and define Natural transform and fractional calculus. In Section 3, the idea of Natural transform decomposition method is discussed. In Section 4, we explain many problems to maintaining the accuracy and efficiency of the proposed method, and Section 5 is devoted to the conclusion.

## 2. Preliminaries

**Definition** **1.** *The natural transform of $g(t_{1})$ is defined as [37,38]*

$$N^{+}\left[g\left(t_{1}\right)\right]=Q\left(s,u\right)=\frac{1}{u}\int_{0}^{\infty }e^{\frac{-st_{1}}{u}}g\left(t_{1}\right)dt_{1};\;s,u>0,$$

*where s and u are the transform variables.*

**Definition** **2.** *The inverse natural transform of a function is defined by*

$$N^{-}\left[Q\left(s,u\right)\right]=g\left(t_{1}\right)=\frac{1}{2\pi i}\int_{p-i\infty }^{p+i\infty }e^{\frac{st_{1}}{u}}Q(s,u)ds,$$

*where s and u are the Natural transform variables, p is a real constant, and the integral is taken along s=p in the complex plane $s=x_{1}+iy_{1}$.*

**Definition** **3.** *Natural Transform of n-th Derivative*

*If $g^{n}\left(t_{1}\right)$ is the n-th derivative of function $g(t_{1})$, it is given by*

$$N\left[g^{n}\left(t_{1}\right)\right]=Q_{n}\left(s,u\right)=\frac{s^{n}}{u^{n}}Q\left(s,u\right)-\sum_{k=0}^{n-1}\frac{s^{n-(k+1)}}{u^{n-k}}g^{k}\left(0\right),\;n\geq 1.$$

**Theorem** **1.** *If H(s,u) and L(s,u) are the transform functions $h(t_{1})$ and $l(t_{1})$, respectively, they are given by,*

N[h∗l]=uH(s,u)L(s,u),

*where h∗l is the convolution of two functions h and l.*

**Definition** **4.** *R–L fractional integral*

$$I_{x_{1}}^{\gamma }g\left(x_{1}\right)=\left\{\begin{array}{cc} g(x_{1}) & \mathrm{if}\;\gamma =0\\ \frac{1}{\Gamma (\gamma )}\int_{0}^{x_{1}}{\left(x_{1}-\upsilon \right)}^{\gamma -1}g\left(\upsilon \right)d\upsilon & \mathrm{if}\;\gamma >0, \end{array}\right.$$

*where *Γ* denotes the gamma function, defined by*

$$\Gamma \left(\omega \right)=\int_{0}^{\infty }e^{-x_{1}}{x_{1}}^{\omega -1}dx_{1}\;\omega \in C.$$

In their study, Caputo et al. suggested a revised fractional derivative operator in order to overcome inconsistencies measured in the Riemann–Liouville derivative. The above mathematical statement described a Caputo fractional derivative operator of initial and boundary condition for fractional—as well as integer-order derivatives [39,40].

**Definition** **5.** *The Caputo operator of order γ for a fractional derivative is given by the following mathematical expression for n∈N, $x_{1}>0$, $g\in C_{t_{1}}$, $t_{1}\geq -1$ [41]:*

$$D^{\gamma }g\left(x_{1}\right)=\frac{\partial^{\gamma }g\left(x_{1}\right)}{\partial t_{1}^{\gamma }}=\left\{\begin{array}{cc} I^{n-\gamma }\left[\frac{\partial^{\gamma }g\left(x_{1}\right)}{\partial t_{1}^{\gamma }}\right], & \mathrm{if}\;n-1<\gamma \leq n,\;n\in N\\ \frac{\partial^{\gamma }g\left(x_{1}\right)}{\partial t_{1}^{\gamma }}. \end{array}\right.$$

**Definition** **6.** *TheMittag–Leffler function $E_{\gamma }\left(p\right)$ for γ>0 is defined by the following subsequent series*

$$E_{\gamma }\left(p\right)=\sum_{n=0}^{\infty }\frac{p^{n}}{\Gamma (\gamma n+1)}\;\gamma >0\;p\in C.$$

**Theorem** **2.** *Here, we will study the convergence analysis, in the same manner as in [42], of the NTDM applied to the fractional dispersive PDE of order three. Let us consider the Hilbert space H, which may defined by $H=L^{2}\left(\left(\alpha ,\beta \right)X\left[0,T\right]\right)$ the set of applications:*

$$u:\left(\alpha ,\beta \right)X\left[0,T\right]\to \;with\;\int_{\left(\alpha ,\beta )X[0,T\right]}u^{2}\left(x,s\right)dsd\theta \;<+\infty .$$

*Now we consider the fractional-order of diffusion equations of order three in the above assumptions which lets us denote*

$$L\left(u\right)=\frac{\partial^{\gamma }u}{\partial t^{\gamma }},$$

*then the fractional-order of diffusion equations becomes, in an operator form,*

$$L\left(u\right)=\varphi \frac{\partial^{2}\upsilon \left(x_{1},t_{1}\right)}{\partial x_{1}^{2}}-w\frac{\partial^{2}\upsilon \left(x_{1},t_{1}\right)}{\partial y_{1}^{2}}.$$

*The NTDM reaches convergence if the following two hypotheses are satisfied:*

$$\left(H1\right)\left(L\left(u\right)-L\left(v\right),u-v\right)\geq k\|u-v{\|}^{2};k>0,\forall u,v\epsilon H.$$

*H(2) whatever may be M>0, there exist a constant C(M)>0 such that for u,vϵH with ‖u‖≤M, ‖v‖≤M we have (L(u)−L(v),u−v)≤C(M)‖u−v‖‖w‖ for every wϵH.*

## 3. Idea of Fractional Natural Transform Decomposition Method

In this section, we use the Natural transform decomposition method to find the general solution fractional-order diffusion equations.

(1) $$D^{\gamma }\upsilon \left(x_{1},t_{1}\right)+L\upsilon \left(x_{1},t_{1}\right)+N\upsilon \left(x_{1},t_{1}\right)=q\left(x_{1},t_{1}\right),\;x_{1},t_{1}\geq 0,\;m-1<\gamma <m,$$

where $D^{\gamma }=\frac{\partial^{\gamma }}{\partial t_{1}^{\gamma }}$ is the Caputo Operator γ,m∈N, L and N are respectively linear and non-linear functions, and *q* is the source function.

The initial condition is

(2) $$\upsilon \left(x_{1},0\right)=k\left(x_{1}\right),\;0<\gamma \leq 1,\;t_{1}>0.$$

Applying the Natural transform to Equation (1), we have

(3) $$N^{+}\left[D^{\gamma }\upsilon \left(x_{1},t_{1}\right)\right]+N^{+}\left[L\upsilon \left(x_{1},t_{1}\right)+N\upsilon \left(x_{1},t_{1}\right)\right]=N^{+}\left[q(x_{1},t_{1})\right],$$

and using the differentiation property of Natural transform, we get

$$\frac{s^{\gamma }}{u^{\gamma }}N^{+}\left[\upsilon (x_{1},t_{1})\right]-\frac{s^{\gamma -1}}{u^{\gamma }}\upsilon \left(x_{1},0\right)=N^{+}\left[q(x_{1},t_{1})\right]-N^{+}\left[L\upsilon \left(x_{1},t_{1}\right)+N\upsilon \left(x_{1},t_{1}\right)\right],$$

$$N^{+}\left[\upsilon (x_{1},t_{1})\right]=\frac{1}{s}\upsilon \left(x_{1},0\right)+\frac{u^{\gamma }}{s^{\gamma }}N^{+}\left[q(x_{1},t_{1})\right]-\frac{u^{\gamma }}{s^{\gamma }}N^{+}\left[L\upsilon \left(x_{1},t_{1}\right)+N\upsilon \left(x_{1},t_{1}\right)\right].$$

Now $\upsilon \left(x_{1},0\right)=k\left(x_{1}\right)$,

(4) $$N^{+}\left[\upsilon (x_{1},t_{1})\right]=\frac{k(x_{1})}{s}+\frac{u^{\gamma }}{s^{\gamma }}N^{+}\left[q(x_{1},t_{1})\right]-\frac{u^{\gamma }}{s^{\gamma }}N^{+}\left[L\upsilon \left(x_{1},t_{1}\right)+N\upsilon \left(x_{1},t_{1}\right)\right].$$

The NTDM solution $\upsilon (x_{1},t_{1})$ is represented by the following infinite series:

(5) $$\upsilon \left(x_{1},t_{1}\right)=\sum_{j=0}^{\infty }\upsilon_{j}\left(x_{1},t_{1}\right),$$

and the non-linear terms (if any) in the problem are defined by the infinite series of Adomian polynomials

(6) $$N\upsilon (x_{1},t_{1})=\sum_{j=0}^{\infty }A_{j},$$

(7) $$A_{j}=\frac{1}{j!}{\left[\frac{d^{j}}{d\lambda^{j}}\left[N\sum_{j=0}^{\infty }\left(\lambda^{j}\upsilon_{j}\right)\right]\right]}_{\lambda =0},\;j=0,1,2\ldots$$

substituting Equations (5) and (6) into Equation (4), we get

(8) $$N^{+}\left[\sum_{j=0}^{\infty }\upsilon_{j}\left(x_{1},t_{1}\right)\right]=\frac{k(x_{1})}{s}+\frac{u^{\gamma }}{s^{\gamma }}N^{+}\left[q(x_{1},t_{1})\right]-\frac{u^{\gamma }}{s^{\gamma }}N^{+}\left[L\sum_{j=0}^{\infty }\upsilon_{j}\left(x_{1},t_{1}\right)+\sum_{j=0}^{\infty }A_{j}\right].$$

Applying the linearity of the Natural transform,

(9) $$N^{+}\left[\upsilon_{0}\left(x_{1},t_{1}\right)\right]=\frac{k(x_{1})}{s}+\frac{u^{\gamma }}{s^{\gamma }}N^{+}\left[q(x_{1},t_{1})\right],$$

$$N^{+}\left[\upsilon_{1}\left(x_{1},t_{1}\right)\right]=-\frac{u^{\gamma }}{s^{\gamma }}N^{+}\left[L\upsilon_{0}\left(x_{1},t_{1}\right)+A_{0}\right].$$

Generally, we can write

(10) $$N^{+}\left[\upsilon_{j+1}\left(x_{1},t_{1}\right)\right]=-\frac{u^{\gamma }}{s^{\gamma }}N^{+}\left[L\upsilon_{j}\left(x_{1},t_{1}\right)+A_{j}\right],\;j\geq 1.$$

Applying the inverse Natural transform, Equations (9) and (10)

$$\upsilon_{0}\left(x_{1},t_{1}\right)=k\left(x_{1}\right)+N^{-}\left[\frac{u^{\gamma }}{s^{\gamma }}N^{+}\left[q(x_{1},t_{1})\right]\right],$$

(11) $$\upsilon_{j+1}\left(x_{1},t_{1}\right)=-N^{-}\left[\frac{u^{\gamma }}{s^{\gamma }}N^{+}\left[L\upsilon_{j}\left(x_{1},t_{1}\right)+A_{j}\right]\right].$$

## 4. Results

### 4.1. Example

Consider the two-dimensional fractional diffusion equation [26]:

(12) $$\frac{\partial^{\gamma }\upsilon }{\partial t_{1}^{\gamma }}=\frac{\partial^{2}\upsilon }{\partial x_{1}^{2}}+\frac{\partial^{2}\upsilon }{\partial y_{1}^{2}},\;0<\gamma \leq 1,\;t_{1}\geq 0,$$

with the initial condition

(13) $$\upsilon \left(x_{1},y_{1},0\right)=\left(1-y_{1}\right)e^{x_{1}}.$$

Taking the Natural transform of Equation (12),

$$\frac{s^{\gamma }}{u^{\gamma }}N^{+}\left[\upsilon (x_{1},y_{1},t_{1})\right]-\frac{s^{\gamma -1}}{u^{\gamma }}\upsilon (x_{1},y_{1},0)=N^{+}\left[\frac{\partial^{2}\upsilon }{\partial x_{1}^{2}}+\frac{\partial^{2}\upsilon }{\partial y_{1}^{2}}\right].$$

Applying inverse Natural transform, we get

$$\upsilon (x_{1},y_{1},t_{1})=N^{-}\left[\frac{\upsilon (x_{1},y_{1},0)}{s}-\frac{u^{\gamma }}{s^{\gamma }}N^{+}\left[\frac{\partial^{2}\upsilon }{\partial x_{1}^{2}}+\frac{\partial^{2}\upsilon }{\partial y_{1}^{2}}\right]\right].$$

Using the ADM procedure, we get

$$\upsilon_{0}\left(x_{1},y_{1},t_{1}\right)=N^{-}\left[\frac{\upsilon (x_{1},y_{1},0)}{s}\right]=N^{-}\left[\frac{\left(1-y_{1}\right)e^{x_{1}}}{s}\right],$$

(14) $$\upsilon_{0}\left(x_{1},y_{1},t_{1}\right)=\left(1-y_{1}\right)e^{x_{1}},$$

$$\upsilon_{j+1}\left(x_{1},y_{1},t_{1}\right)=N^{-}\left[\frac{u^{\gamma }}{s^{\gamma }}N^{+}\left[\frac{\partial^{2}\upsilon_{j}}{\partial x_{1}^{2}}+\frac{\partial^{2}\upsilon_{j}}{\partial y_{1}^{2}}\right]\right],\;j=0,1,2,\ldots$$

for j=0:

(15) $$\begin{array}{c} \upsilon_{1}\left(x_{1},y_{1},t_{1}\right)=N^{-}\left[\frac{u^{\gamma }}{s^{\gamma }}N^{+}\left[\frac{\partial^{2}\upsilon_{0}}{\partial x_{1}^{2}}+\frac{\partial^{2}\upsilon_{0}}{\partial y_{1}^{2}}\right]\right],\\ \upsilon_{1}\left(x_{1},y_{1},t_{1}\right)=N^{-}\left[\frac{\left(1-y_{1}\right)e^{x_{1}}u^{\gamma }}{s^{\gamma +1}}\right]=\left(1-y_{1}\right)e^{x_{1}}\frac{t_{1}^{\gamma }}{\Gamma (\gamma +1)}. \end{array}$$

The subsequent terms are

(16) $$\begin{array}{c} \upsilon_{2}\left(x_{1},y_{1},t_{1}\right)=N^{-}\left[\frac{u^{\gamma }}{s^{\gamma }}N^{+}\left[\frac{\partial^{2}\upsilon_{1}}{\partial x_{1}^{2}}+\frac{\partial^{2}\upsilon_{1}}{\partial y_{1}^{2}}\right]\right]=\left(1-y_{1}\right)e^{x_{1}}\frac{t_{1}^{2\gamma }}{\Gamma (2\gamma +1)},\\ \upsilon_{3}\left(x_{1},y_{1},t_{1}\right)=N^{-}\left[\frac{u^{\gamma }}{s^{\gamma }}N^{+}\left[\frac{\partial^{2}\upsilon_{2}}{\partial x_{1}^{2}}+\frac{\partial^{2}\upsilon_{2}}{\partial y_{1}^{2}}\right]\right]=\left(1-y_{1}\right)e^{x_{1}}\frac{t_{1}^{3\gamma }}{\Gamma (3\gamma +1)},\\ \upsilon_{3}\left(x_{1},y_{1},t_{1}\right)=N^{-}\left[\frac{u^{\gamma }}{s^{\gamma }}N^{+}\left[\frac{\partial^{2}\upsilon_{3}}{\partial x_{1}^{2}}+\frac{\partial^{2}\upsilon_{3}}{\partial y_{1}^{2}}\right]\right]=\left(1-y_{1}\right)e^{x_{1}}\frac{t_{1}^{4\gamma }}{\Gamma (4\gamma +1)}\cdot \\ .\\ . \end{array}$$

The NTDM solution for Example 4.1 is:

$$\upsilon \left(x_{1},y_{1},t_{1}\right)=\upsilon_{0}\left(x_{1},y_{1},t_{1}\right)+\upsilon_{1}\left(x_{1},y_{1},t_{1}\right)+\upsilon_{2}\left(x_{1},y_{1},t_{1}\right)+\upsilon_{3}\left(x_{1},y_{1},t_{1}\right)+\upsilon_{4}\left(x_{1},y_{1},t_{1}\right)\ldots$$

$$\upsilon \left(x_{1},y_{1},t_{1}\right)=\left(1-y_{1}\right)e^{x_{1}}\left(1+\frac{t_{1}^{\gamma }}{\Gamma (\gamma +1)}+\frac{t_{1}^{2\gamma }}{\Gamma (2\gamma +1)}+\frac{t_{1}^{3\gamma }}{\Gamma (3\gamma +1)}+\frac{t_{1}^{4\gamma }}{\Gamma (4\gamma +1)}\ldots \right).$$

When γ=1, the NTDM solution is

(17) $$\upsilon \left(x_{1},y_{1},t_{1}\right)=\left(1-y_{1}\right)e^{x_{1}}\left(1+t_{1}+\frac{t_{1}^{2}}{2!}+\frac{t_{1}^{3}}{3!}+\frac{t_{1}^{4}}{4!}\ldots \right).$$

This result is calculated to the exact solution in a closed form:

$$\upsilon \left(x_{1},y_{1},t_{1}\right)=\left(1-y_{1}\right)e^{x_{1}+t_{1}}.$$

**Figure 1:**
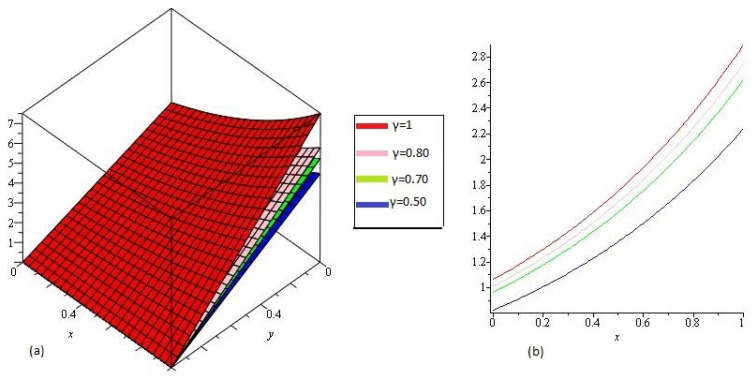
The (**a**) NTDM of $\upsilon (x_{1},y_{1},t_{1})$ of Example 4.1, for different value of γ and (**b**) y=0.5.

In Figure 1 NTDM solution of Example 4.1 of different value of γ=1, 0.80, 0.70 and 0.50 and 0<x,y≤1 are represented by Figure 1a and Figure 1b respectively at y=1, tϵ[0,1] and 0<x≤1. From the given graphs it can be observed that both exact and NTMD solutions are in strong agrement with each other.

### 4.2. Example

Consider the two-dimensional fractional diffusion equation [26]:

(18) $$\frac{\partial^{\gamma }\upsilon }{\partial t_{1}^{\gamma }}=\frac{\partial^{2}\upsilon }{\partial x_{1}^{2}}+\frac{\partial^{2}\upsilon }{\partial y_{1}^{2}},\;0<\gamma \leq 1,\;t_{1}\geq 0,$$

with the initial condition

(19) $$\upsilon \left(x_{1},y_{1},0\right)=e^{x_{1}+y_{1}}.$$

Taking the Natural transform of Equation (18):

$$\frac{s^{\gamma }}{u^{\gamma }}N^{+}\left[\upsilon (x_{1},y_{1},t_{1})\right]-\frac{s^{\gamma -1}}{u^{\gamma }}\upsilon (x_{1},y_{1},0)=N^{+}\left[\frac{\partial^{2}\upsilon }{\partial x_{1}^{2}}+\frac{\partial^{2}\upsilon }{\partial y_{1}^{2}}\right].$$

Applying inverse Natural transform, we get

$$\upsilon (x_{1},y_{1},t_{1})=N^{-}\left[\frac{\upsilon (x_{1},y_{1},0)}{s}-\frac{u^{\gamma }}{s^{\gamma }}N^{+}\left[\frac{\partial^{2}\upsilon }{\partial x_{1}^{2}}+\frac{\partial^{2}\upsilon }{\partial y_{1}^{2}}\right]\right].$$

Using ADM procedure, we get

$$\upsilon_{0}\left(x_{1},y_{1},t_{1}\right)=N^{-}\left[\frac{\upsilon (x_{1},y_{1},0)}{s}\right]=N^{-}\left[\frac{e^{x_{1}+y_{1}}}{s}\right],$$

(20) $$\upsilon_{0}\left(x_{1},y_{1},t_{1}\right)=e^{x_{1}+y_{1}},$$

$$\upsilon_{j+1}\left(x_{1},y_{1},t_{1}\right)=N^{-}\left[\frac{u^{\gamma }}{s^{\gamma }}N^{+}\left[\frac{\partial^{2}\upsilon_{j}}{\partial x_{1}^{2}}+\frac{\partial^{2}\upsilon_{j}}{\partial y_{1}^{2}}\right]\right],\;j=0,1,2,\ldots$$

for j=0:

(21) $$\begin{array}{c} \upsilon_{1}\left(x_{1},y_{1},t_{1}\right)=N^{-}\left[\frac{u^{\gamma }}{s^{\gamma }}N^{+}\left[\frac{\partial^{2}\upsilon_{0}}{\partial x_{1}^{2}}+\frac{\partial^{2}\upsilon_{0}}{\partial y_{1}^{2}}\right]\right],\\ \upsilon_{1}\left(x_{1},y_{1},t_{1}\right)=N^{-}\left[\frac{2e^{x_{1}+y_{1}}u^{\gamma }}{s^{\gamma +1}}\right]=2e^{x_{1}+y_{1}}\frac{t_{1}^{\gamma }}{\Gamma (\gamma +1)}. \end{array}$$

(22) $$\begin{array}{c} \upsilon_{2}\left(x_{1},y_{1},t_{1}\right)=N^{-}\left[\frac{u^{\gamma }}{s^{\gamma }}N^{+}\left[\frac{\partial^{2}\upsilon_{1}}{\partial x_{1}^{2}}+\frac{\partial^{2}\upsilon_{1}}{\partial y_{1}^{2}}\right]\right]=4e^{x_{1}+y_{1}}\frac{t_{1}^{2\gamma }}{\Gamma (2\gamma +1)},\\ \upsilon_{3}\left(x_{1},y_{1},t_{1}\right)=N^{-}\left[\frac{u^{\gamma }}{s^{\gamma }}N^{+}\left[\frac{\partial^{2}\upsilon_{2}}{\partial x_{1}^{2}}+\frac{\partial^{2}\upsilon_{2}}{\partial y_{1}^{2}}\right]\right]=8e^{x_{1}+y_{1}}\frac{t_{1}^{3\gamma }}{\Gamma (3\gamma +1)},\\ \upsilon_{3}\left(x_{1},y_{1},t_{1}\right)=N^{-}\left[\frac{u^{\gamma }}{s^{\gamma }}N^{+}\left[\frac{\partial^{2}\upsilon_{3}}{\partial x_{1}^{2}}+\frac{\partial^{2}\upsilon_{3}}{\partial y_{1}^{2}}\right]\right]=16e^{x_{1}+y_{1}}\frac{t_{1}^{4\gamma }}{\Gamma (4\gamma +1)}.\\ .\\ . \end{array}$$

The NTDM solution for Example 4.2 is

$$\upsilon \left(x_{1},y_{1},t_{1}\right)=\upsilon_{0}\left(x_{1},y_{1},t_{1}\right)+\upsilon_{1}\left(x_{1},y_{1},t_{1}\right)+\upsilon_{2}\left(x_{1},y_{1},t_{1}\right)+\upsilon_{3}\left(x_{1},y_{1},t_{1}\right)+\upsilon_{4}\left(x_{1},y_{1},t_{1}\right)\ldots$$

$$\upsilon \left(x_{1},y_{1},t_{1}\right)=e^{x_{1}+y_{1}}\left(1+\frac{t_{1}^{\gamma }}{\Gamma (\gamma +1)}+\frac{{\left(2t_{1}^{\gamma }\right)}^{2}}{\Gamma (2\gamma +1)}+\frac{{\left(2t_{1}^{\gamma }\right)}^{3}}{\Gamma (3\gamma +1)}+\frac{{\left(2t_{1}^{\gamma }\right)}^{4}}{\Gamma (4\gamma +1)}\ldots \right).$$

When γ=1, then the NTDM solution is

(23) $$\upsilon \left(x_{1},y_{1},t_{1}\right)=e^{x_{1}+y_{1}}\left(1+2t_{1}+\frac{{\left(2t_{1}\right)}^{2}}{2!}+\frac{{\left(2t_{1}\right)}^{3}}{3!}+\frac{{\left(2t_{1}\right)}^{4}}{4!}\ldots \right).$$

This result is calculated to the exact solution in a closed form:

$$\upsilon \left(x_{1},y_{1},t_{1}\right)=e^{x_{1}+y_{1}+t_{1}}.$$

**Figure 2:**
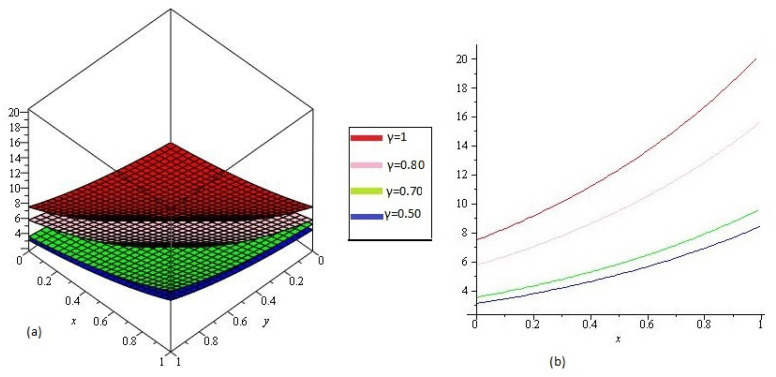
The (**a**) NTDM solutions of $\upsilon (x_{1},y_{1},t_{1})$ of Example 4.2, for different values of γ and (**b**) y=1.

In Figure 2 NTDM solution of Example 4.2 of different value of γ=1, 0.80, 0.70 and 0.50 and 0<x,y≤1 are represented by Figure 2a and Figure 2b respectively at y=1, tϵ[0,1] and 0<x≤1. From the given graphs it can be observed that both exact and NTDM solutions are in strong agrement with each other.

### 4.3. Example

Consider the three-dimensional fractional diffusion equation [25]:

(24) $$\frac{\partial^{\gamma }\upsilon }{\partial t_{1}^{\gamma }}=\frac{\partial^{2}\upsilon }{\partial x_{1}^{2}}+\frac{\partial^{2}\upsilon }{\partial y_{1}^{2}}+\frac{\partial^{2}\upsilon }{\partial z_{1}^{2}},\;0<\gamma \leq 1,\;t_{1}\geq 0,$$

with the initial condition

(25) $$\upsilon \left(x_{1},y_{1},z_{1},0\right)=\sin x_{1}\sin y_{1}\sin z_{1}.$$

Taking the Natural transform of Equation (24),

$$\frac{s^{\gamma }}{u^{\gamma }}N^{+}\left[\upsilon (x_{1},y_{1},z_{1},t_{1})\right]-\frac{s^{\gamma -1}}{u^{\gamma }}\upsilon (x_{1},y_{1},z_{1},0)=N^{+}\left[\frac{\partial^{2}\upsilon }{\partial x_{1}^{2}}+\frac{\partial^{2}\upsilon }{\partial y_{1}^{2}}+\frac{\partial^{2}\upsilon }{\partial z_{1}^{2}}\right].$$

Applying inverse Natural transform, we get

$$\upsilon (x_{1},y_{1},z_{1},t_{1})=N^{-}\left[\frac{\upsilon (x_{1},y_{1},z_{1},0)}{s}-\frac{u^{\gamma }}{s^{\gamma }}N^{+}\left[\frac{\partial^{2}\upsilon }{\partial x_{1}^{2}}+\frac{\partial^{2}\upsilon }{\partial y_{1}^{2}}+\frac{\partial^{2}\upsilon }{\partial z_{1}^{2}}\right]\right].$$

Using ADM procedure, we get

$$\upsilon_{0}\left(x_{1},y_{1},z_{1},t_{1}\right)=N^{-}\left[\frac{\upsilon (x_{1},y_{1},z_{1},0)}{s}\right]=N^{-}\left[\frac{\sin x_{1}\sin y_{1}\sin z_{1}}{s}\right],$$

(26) $$\upsilon_{0}\left(x_{1},y_{1},z_{1},t_{1}\right)=\sin x_{1}\sin y_{1}\sin z_{1},$$

$$\upsilon_{j+1}\left(x_{1},y_{1},z_{1},t_{1}\right)=N^{-}\left[\frac{u^{\gamma }}{s^{\gamma }}N^{+}\left[\frac{\partial^{2}\upsilon_{j}}{\partial x_{1}^{2}}+\frac{\partial^{2}\upsilon_{j}}{\partial y_{1}^{2}}+\frac{\partial^{2}\upsilon_{j}}{\partial z_{1}^{2}}\right]\right],\;j=0,1,2,\ldots$$

for j=0:

(27) $$\begin{array}{c} \upsilon_{1}\left(x_{1},y_{1},z_{1},t_{1}\right)=N^{-}\left[\frac{u^{\gamma }}{s^{\gamma }}N^{+}\left[\frac{\partial^{2}\upsilon_{0}}{\partial x_{1}^{2}}+\frac{\partial^{2}\upsilon_{0}}{\partial y_{1}^{2}}+\frac{\partial^{2}\upsilon_{0}}{\partial z_{1}^{2}}\right]\right],\\ \upsilon_{1}\left(x_{1},y_{1},z_{1},t_{1}\right)=N^{-}\left[\frac{2\sin x_{1}\sin y_{1}\sin z_{1}u^{\gamma }}{s^{\gamma +1}}\right]=-3\sin x_{1}\sin y_{1}\sin z_{1}\frac{t_{1}^{\gamma }}{\Gamma (\gamma +1)}. \end{array}$$

The subsequent terms are:

(28) $$\begin{array}{c} \upsilon_{2}\left(x_{1},y_{1},z_{1},t_{1}\right)=N^{-}\left[\frac{u^{\gamma }}{s^{\gamma }}N^{+}\left[\frac{\partial^{2}\upsilon_{1}}{\partial x_{1}^{2}}+\frac{\partial^{2}\upsilon_{1}}{\partial y_{1}^{2}}+\frac{\partial^{2}\upsilon_{1}}{\partial z_{1}^{2}}\right]\right]={\left(-3\right)}^{2}\sin x_{1}\sin y_{1}\sin z_{1}\frac{t_{1}^{2\gamma }}{\Gamma (2\gamma +1)},\\ \upsilon_{3}\left(x_{1},y_{1},z_{1},t_{1}\right)=N^{-}\left[\frac{u^{\gamma }}{s^{\gamma }}N^{+}\left[\frac{\partial^{2}\upsilon_{2}}{\partial x_{1}^{2}}+\frac{\partial^{2}\upsilon_{2}}{\partial y_{1}^{2}}+\frac{\partial^{2}\upsilon_{2}}{\partial z_{1}^{2}}\right]\right]={\left(-3\right)}^{3}\sin x_{1}\sin y_{1}\sin z_{1}\frac{t_{1}^{3\gamma }}{\Gamma (3\gamma +1)},\\ \upsilon_{3}\left(x_{1},y_{1},z_{1},t_{1}\right)=N^{-}\left[\frac{u^{\gamma }}{s^{\gamma }}N^{+}\left[\frac{\partial^{2}\upsilon_{3}}{\partial x_{1}^{2}}+\frac{\partial^{2}\upsilon_{3}}{\partial y_{1}^{2}}+\frac{\partial^{2}\upsilon_{3}}{\partial z_{1}^{2}}\right]\right]={\left(-3\right)}^{4}\sin x_{1}\sin y_{1}\sin z_{1}\frac{t_{1}^{4\gamma }}{\Gamma (4\gamma +1)},\\ .\\ .\\ .\\ \upsilon_{j}\left(x_{1},y_{1},z_{1},t_{1}\right)=N^{-}\left[\frac{u^{\gamma }}{s^{\gamma }}N^{+}\left[\frac{\partial^{2}\upsilon_{j}}{\partial x_{1}^{2}}+\frac{\partial^{2}\upsilon_{j}}{\partial y_{1}^{2}}+\frac{\partial^{2}\upsilon_{j}}{\partial z_{1}^{2}}\right]\right]={\left(-3\right)}^{j}\sin x_{1}\sin y_{1}\sin z_{1}\frac{t_{1}^{j\gamma }}{\Gamma (j\gamma +1)}. \end{array}$$

The NTDM solution for Example 4.3 is

$$\upsilon \left(x_{1},y_{1},z_{1},t_{1}\right)=\upsilon_{0}\left(x_{1},y_{1},z_{1},t_{1}\right)+\upsilon_{1}\left(x_{1},y_{1},z_{1},t_{1}\right)+\upsilon_{2}\left(x_{1},y_{1},z_{1},t_{1}\right)+\upsilon_{3}\left(x_{1},y_{1},z_{1},t_{1}\right)+\ldots$$

$$\upsilon \left(x_{1},y_{1},z_{1},t_{1}\right)=\sin x_{1}\sin y_{1}\sin z_{1}\left(1-\frac{3t_{1}^{\gamma }}{\Gamma (\gamma +1)}+\frac{{\left(-3t_{1}^{\gamma }\right)}^{2}}{\Gamma (2\gamma +1)}+\frac{{\left(-3t_{1}^{\gamma }\right)}^{3}}{\Gamma (3\gamma +1)}+\frac{{\left(-3t_{1}^{\gamma }\right)}^{4}}{\Gamma (4\gamma +1)}\ldots \right).$$

When γ=1, then the NTDM solution is

(29) $$\upsilon \left(x_{1},y_{1},z_{1},t_{1}\right)=\sin x_{1}\sin y_{1}\sin z_{1}\left(1-3t_{1}+\frac{{\left(-3t_{1}\right)}^{2}}{2!}+\frac{{\left(-3t_{1}\right)}^{3}}{3!}+\frac{{\left(-3t_{1}\right)}^{4}}{4!}\ldots \right).$$

This result is calculated to the exact solution in a closed form:

$$\upsilon \left(x_{1},y_{1},z_{1},t_{1}\right)=e^{-3t_{1}}\sin x_{1}\sin y_{1}\sin z_{1}.$$

**Figure 3:**
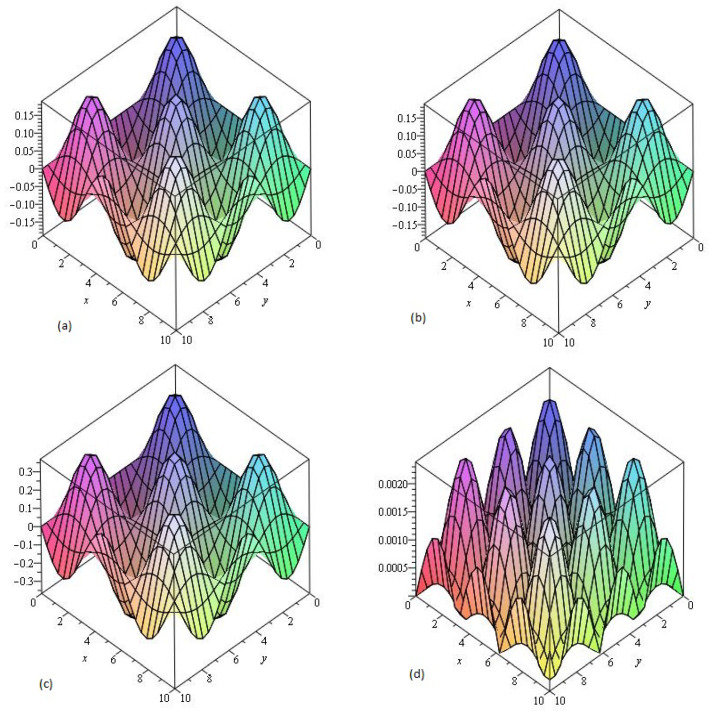
The (**a**) Exact and (**b**) NTDM solutions of $\upsilon (x_{1},y_{1},z_{1},t_{1})$ of Example 4.3, at γ=1, (**c**) at γ=0.50 and (**d**) Error plot at γ=1.

Similarly, in Figure 3 the numerical values of the Example 4.3 show the accuracy and efficiency of the NTDM at different values of γ. In Figure 3a,b we consider fixed order γ=1 for piecewise approximation values of $x_{1},y_{1}$ in the domain $0\leq x_{1},y_{1}\leq 10$. Figure 3c represents the graphs of NTDM solution at γ=0.50, and error Figure 3d at γ=1 respectively of Example 4.3. It is cleared from the Figure 3a,b that NTDM solution are in good agreement with the exact solution of the problems. The small difference from the solutions graph of the problem, because the solution of the fractional-order problems creates a little deviation from the solution at integer order problem.

## 5. Conclusions

In this paper, the analytical solutions of fractional-order diffusion equations are determined, using NTDM. The NTDM solutions are obtained at fractional and integer orders for all problems. The results revealed the highest agreement with the exact solutions for the problems. The NTDM solutions for some numerical examples have shown the validity of the proposed method. It is also investigated that the fractional-order solutions are convergent to the exact solution for the problems as fractional-order approaches integer-order. The implementation of NTDM to illustrative examples have also confirmed that the fractional-order mathematical model can be the best representation of any experimental data compared to the integer-order model. Moreover, by taking different fractional orders, we can find a way to set a suitable mathematical model for any experimental data, and thus find reasonable consequences. Hence, it is concluded that NTDM is the best tool for the solution of FPDEs compared to ADM, VIM, and DTM discussed in literature. NTDM provides the highest rate of convergence to the exact solution for the problems. In the future, NTDM can be used to find the analytical solution of other non-linear FPDEs, which are frequently used in science and engineering. NTDM solutions for fractional-order problems will prove the best understanding of the real world problems represented by FPDEs.

## References

[1] Gómez-Aguilar J.F., Atangana A. (2017). Fractional Hunter-Saxton equation involving partial operators with bi-order in Riemann-Liouville and Liouville-Caputo sense. Eur. Phys. J. Plus.

[2] Arshad S., Baleanu D., Huang J., Al Qurashi M., Tang Y., Zhao Y. (2018). Finite Difference Method for Time-Space Fractional Advection–Diffusion Equations with Riesz Derivative. Entropy.

[3] Machado J.A.T. (2010). Entropy analysis of integer and fractional dynamical systems. Nonlinear Dyn..

[4] Hoffmann K.H., Essex C., Schulzky C. (1998). Fractional diffusion and entropy production. J. Non-Equilib. Thermodyn..

[5] Magin R.L., Ingo C. (2012). Entropy and information in a fractional order model of anomalous diffusion. IFAC Proc..

[6] Ubriaco M.R. (2009). Entropies based on fractional calculus. Phys. Lett..

[7] Yépez-Martínez H., Gómez-Aguilar F., Sosa I.O., Reyes J.M., Torres-Jiménez J. (2016). The Feng’s first integral method applied to the nonlinear mKdV space-time fractional partial differential equation. Rev. Mex. Fís..

[8] Ball J.M., Chen G.Q.G. (2013). Entropy and convexity for nonlinear partial differential equations. Philos. Trans. R. Soc. Math. Phys. Eng. Sci..

[9] Shah R., Khan H., Arif M., Kumam P. (2019). Application of Laplace-Adomian Decomposition Method for the Analytical Solution of Third-Order Dispersive Fractional Partial Differential Equations. Entropy.

[10] Sibatov R., Shulezhko V., Svetukhin V. (2017). Fractional Derivative Phenomenology of Percolative Phonon-Assisted Hopping in Two-Dimensional Disordered Systems. Entropy.

[11] Jiang J., Feng Y., Li S. (2018). Exact Solutions to the Fractional Differential Equations with Mixed Partial Derivatives. Axioms.

[12] Prehl J., Essex C., Hoffmann K.H. (2012). Tsallis relative entropy and anomalous diffusion. Entropy.

[13] Cuahutenango-Barro B., Taneco-Hernández M.A., Gómez-Aguilar J.F. (2018). On the solutions of fractional-time wave equation with memory effect involving operators with regular kernel. Chaos Solitons Fractals.

[14] Gómez-Gardeñes J., Latora V. (2008). Entropy rate of diffusion processes on complex networks. Phys. Rev..

[15] Lopes A.M., Tenreiro Machado J.A. (2019). Entropy Analysis of Soccer Dynamics. Entropy.

[16] Bejan A. (1982). Second-law analysis in heat transfer and thermal design. Adv. Heat Transf..

[17] Bejan A. (1979). A study of entropy generation in fundamental convective heat transfer. J. Heat Transf..

[18] Syam M., Al-Refai M. (2014). Solving fractional diffusion equation via the collocation method based on fractional legendre functions. Comput. Methods Phys..

[19] Lenzi E., dos Santos M., Michels F., Mendes R., Evangelista L. (2013). Solutions of some nonlinear diffusion equations and generalized entropy framework. Entropy.

[20] Prehl J., Boldt F., Hoffmann K., Essex C. (2016). Symmetric fractional diffusion and entropy production. Entropy.

[21] Dehghan M., Abbaszadeh M. (2018). A finite difference/finite element technique with error estimate for space fractional tempered diffusion-wave equation. Comput. Math. Appl..

[22] Lei S.L., Huang Y.C. (2017). Fast algorithms for high-order numerical methods for space-fractional diffusion equations. Int. J. Comput. Math..

[23] Sepahvandzadeh A., Ghazanfari B., Asadian N. (2018). Numerical Solution of Stochastic Generalized Fractional Diffusion Equation by Finite Difference Method. Math. Comput. Appl..

[24] Tripathi N., Das S., Ong S., Jafari H., Al Qurashi M. (2016). Solution of higher order nonlinear time-fractional reaction diffusion equation. Entropy.

[25] Shah K., Khalil H., Khan R.A. (2016). Analytical solutions of fractional order diffusion equations by natural transform method. Iran. J. Sci. Technol. Trans. Sci..

[26] Kumar D., Singh J., Kumar S. (2015). Numerical computation of fractional multi-dimensional diffusion equations by using a modified homotopy perturbation method. J. Assoc. Arab. Univ. Basic Appl. Sci..

[27] Zafarghandi F.S., Mohammadi M., Babolian E., Javadi S. (2019). Radial basis functions method for solving the fractional diffusion equations. Appl. Math. Comput..

[28] Luchko Y. (2016). Entropy production rate of a one-dimensional alpha-fractional diffusion process. Axioms.

[29] Wei S., Chen W., Zhang Y., Wei H., Garrard R.M. (2018). A local radial basis function collocation method to solve the variable-order time fractional diffusion equation in a two-dimensional irregular domain. Numer. Methods Partial. Differ. Equ..

[30] Das S. (2009). Analytical solution of a fractional diffusion equation by variational iteration method. Comput. Math. Appl..

[31] Rawashdeh M.S., Maitama S. (2014). Solving coupled system of nonlinear PDE’s using the natural decomposition method. Int. J. Pure Appl. Math..

[32] Rawashdeh M.S., Maitama S. (2015). Solving nonlinear ordinary differential equations using the NDM. J. Appl. Anal. Comput..

[33] Rawashdeh M., Maitama S. (2017). Finding exact solutions of nonlinear PDEs using the natural decomposition method. Math. Methods Appl. Sci..

[34] Cherif M.H., Ziane D., Belghaba K. (2018). Fractional natural decomposition method for solving fractional system of nonlinear equations of unsteady flow of a polytropic gas. Nonlinear Stud..

[35] Eltayeb H., Abdalla Y.T., Bachar I., Khabir M.H. (2019). Fractional Telegraph Equation and Its Solution by Natural Transform Decomposition Method. Symmetry.

[36] Abdel-Rady A.S., Rida S.Z., Arafa A.A.M., Abedl-Rahim H.R. (2015). Natural transform for solving fractional models. J. Appl. Math. Phys..

[37] Belgacem F.B.M., Silambarasan R. (2012). November. Advances in the natural transform. AIP Conf. Proc..

[38] Khan Z.H., Khan W.A. (2008). N-transform properties and applications. NUST J. Eng. Sci..

[39] Hilfer R. (2000). Applications of Fractional Calculus in Physics.

[40] Podlubny I. (1998). Fractional Differential Equations: An Introduction to Fractional Derivatives, Fractional Differential Equations, to Methods of Their Solution and Some of Their Applications.

[41] Miller K.S., Ross B. (1993). An Introduction to the Fractional Calculus AND Fractional Differential Equations.

[42] Naghipour A., Manafian J. (2015). Application of the Laplace Adomian decomposition and implicit methods for solving Burgers’ equation. TWMS J. Pure Appl. Math..

